# Evaluating Peer Online Forums to Support Health: Ethical and Practical Challenges

**DOI:** 10.2196/73427

**Published:** 2025-12-24

**Authors:** Fiona Lobban, Neil Caton, Zoe Glossop, Jade Haines, Gemma Hayward, Connor Heapy, Rose Johnston, Steve Jones, Chris Lodge, Karen Machin, Paul Marshall, Tamara Rakic, Paul Rayson, Heather Robinson, Elena Semino, John Vidler

**Affiliations:** 1 Spectrum Centre for Mental Health Research, Division of Health Research, Faculty of Health and Medicine, Lancaster University, HiOne Building, Fisher Drive, Lancaster, LA1 4AT, United Kingdom, 44 0152452218; 2 IT Corporate Services, Berkshire Healthcare NHS Foundation Trust, Bracknell, United Kingdom; 3 IM&T Division, Berkshire Healthcare NHS Foundation Trust, Bracknell, United Kingdom; 4 Independent Researcher, St Helens, United Kingdom; 5 School of Computing and Communications, Lancaster University, Lancaster, United Kingdom; 6 Linguistics and English Language, Lancaster University, Lancaster, United Kingdom

**Keywords:** ethics, research design, online data, health, forums

## Abstract

Many people use peer online forums to seek support for health-related problems. More research is needed to understand the impacts of forum use and how these are generated. However, there are significant ethical and practical challenges with the methods available to do the required research. We examine the key challenges associated with conducting each of the most commonly used online data collection methods: surveys, interviews, forum post analysis, and triangulation of these methods. Based on our learning from the Improving Peer Online Forums (iPOF) study, an interdisciplinary realist-informed mixed methods evaluation of peer online forums, we outline strategies that can be used to address key issues pertaining to assessing important outcomes, facilitating participation, validating participants (users who consent to take part in one or more parts of the study), protecting anonymity, gaining consent, managing risk, multistakeholder engagement, and triangulation. We share this learning to support researchers, reviewers, and ethics committees faced with deciding how best to address these challenges. We highlight the need for open, transparent discussion to ensure the research field keeps pace with evolving technology design and societal attitudes to online data use.

## Introduction

Many people use peer online forums to seek support for health problems. Forums allow users (people who read or post on a forum) to engage in anonymous, asynchronous, text-based communication with those who share similar experiences. Current research suggests a wide range of impacts from using forums, but little understanding of why these impacts vary across forum designs and for different people. More research is needed to understand how forums work to inform decisions around whether they should be funded, promoted, and used by individuals. However, such studies require developing a detailed ethical framework and navigating many practical challenges in applying this. How data are collected and used impacts not only the validity of the research but also directly on how safe forum users feel. If users feel their data are being used for purposes that they do not agree with, fully understand, or have consented to, then this could leave them feeling unsafe and ultimately lead them to leave the forum. Conversely, if users feel their data are being used for purposes they agree with, fully understand, and have consented to, they will feel safe to use the forum. Most previous research in this area has used either online surveys (eg, [1]), user interviews (eg, [2]), or analysis of forum posts (eg, [3]), often in one specific forum. Each method raises ethical and practical challenges, but published discussion of these is limited, often to a brief statement to confirm ethical approval or, that in the case of forum post analysis, the data are already in the public domain with no expectation of privacy, and therefore no ethical review is needed. Both positions are problematic. Ethics committees and institutional governance bodies can ensure that research is conducted in accordance with overarching ethical principles (eg, the Declaration of Helsinki 1964 [4]), and relevant national and international laws (eg, General Data Protection Regulation [GDPR] [5]; and the Online Safety Act in the United Kingdom [6]; California Consumer Privacy Act [CCPA] [7]), but rarely do they include experts in online technological advances, or societal concerns. For example, while open online forum posts may be publicly available, they are generally assumed by users to be nonidentifiable and offered in a specific context, for a particular purpose. When posts are taken out of this context and used for research, their meaning changes, and users no longer have the option to edit or remove their own data. If posts are quoted in research outputs, they may then be used for purposes with which the user disagrees. The user, who thought they were anonymous, may also be identifiable through reverse internet searching [8].

Drawing on the Improving Peer Online Forums (iPOF) study [9] as a case study, we share learning on key issues relevant to developing an ethical framework for studies evaluating online forums or collecting and analyzing online personal data more broadly. We hope this will help guide future researchers, ethical committees, and funding bodies. We refrain from proffering definitive guidelines because we agree with Gliniecka [10] who argues for a “situated ethics approach” to researching online spaces, that is flexible, relational, contextual, responsive to change, and informed by the forum users. As such, each study needs to refine its own ethical framework.

See iPOF case study details in Textbox 1.

Textbox 1. **iPOF case study.**

The iPOF study is a realist-informed mixed methods evaluation of multiple UK mental health forums, provided by health care services, charities, or commercial organizations. The aim was to develop a program theory to explain how online mental health forums impact users’ mental health and well-being and to use this theory to develop best practice tools to improve uptake, safety, and usefulness of online forums [9].

In developing an ethical framework, we worked closely across our multidisciplinary research team and our forum partners. We drew on existing guidelines and frameworks including The UK Government Data Ethics framework [11], the British Psychological Society Ethical Guidelines for Internet-Mediated Research [12], and the Ethical Guidelines for the Association of Internet Research [13]. We worked closely with our NHS host Trust Information Governance Lead to gain Trust Research and Development (R&D) approval and Lancaster University Information Officer (MA) to write a Data Protection Impact Assessment (DPIA) [14] for sponsorship approval.

We designed the study to be as transparent as possible. At the outset, we published an open-access detailed protocol paper [9], our realist synthesis [15], our ethical framework including how data will be managed [16], and our statistical analysis protocol [17]. We promoted the study in open online forums and invited people to ask questions and comment. This ensured that participants consenting to the study were making informed decisions, and that other forum users who chose not to take part could access this information.

The iPOF study was funded by the National Institute for Health and Care Research (NIHR), hosted by Berkshire NHS Foundation Trust in the United Kingdom and conducted in collaboration with researchers at Lancaster University and the University of Manchester.

## Online Forum Evaluation: Study Design

Randomized controlled trials are often cited as the gold standard of health technology assessment [18], but are not well-suited to evaluating existing peer online health forums. Access to existing forums cannot be controlled without severely disrupting the ecological validity of the forum, and identifying a valid control group is problematic. Further, given the range of forum types and diversity of possible intended and unintended impacts already reported in the literature [15], it is unlikely that forums either “work” or do not “work,” so identifying a single primary outcome is often a challenge. Attempts have been made to conduct randomized controlled trials in which online forums have been set up primarily for the study (eg, [19]), but establishing a functioning forum community takes a long time and is challenging and expensive [20].

Realist evaluations offer an alternative and potentially more suitable approach to evaluating forums already flourishing [21]. Realist evaluation is grounded in realist philosophy and starts by eliciting a program theory about how an intervention works. This theory is then tested and refined iteratively, drawing on observational data methods, to develop a deep understanding of what works for whom, why, and in what context. However, these observational studies have their own ethical and practical challenges.

See iPOF study design details in Textbox 2.

Textbox 2. **iPOF study design.**

We wanted to study how forums work in the real world, while minimizing any impact of the research on the functioning of the forum community. We chose a realist-informed multiple case series design in which we recruited 7 forums, purposively sampled for diversity across forum hosts (organization responsible for the platform), target population, forum design (including level and nature of moderation, whether they require registered login, and notification systems), and size and engagement level of population. Forums were identified using snowballing techniques and online searching.

We developed a program theory about how forums work based on previous literature and interviews with key stakeholders, exploring intended and unintended positive and negative impacts, and understanding how different forum designs might work differently for different users. We then tested these theories using a combination of surveys, interviews, and qualitative analysis of forum posts.

Each participating forum signed a collaboration agreement outlining how they would work as equal partners in the research to ensure forum users’ views were prioritized throughout. This required: (1) forum hosts to be actively involved in the research process and design decisions, including engaging in host collaboration meetings, and promoting data collection and involvement in a Patient Public Involvement (PPI) and co-design group to forum users; and (2) to only share forum data that were either openly available to the public with no expectation of privacy, or consent had been freely given to share data for research purposes by users at sign-up. Freely given consent was defined as users having the option to share data but this not being a requirement for forum use.

Forums were paid by the research funder for time spent in research activity in accordance with NIHR guidance [22] but no money was paid to generate a profit from the sharing of data. Forums were deidentified using bird names and described in relation to contextual factors identified in the realist synthesis. The study was overseen by an independent Study Steering Group, including methodological and lived experience experts.

## Data Collection

The main methods used to understand user experiences in online forums include online surveys, interviews, and computational, linguistic, or qualitative analysis of forum posts. Each of these methods has limitations and ethical and practical challenges. Triangulating data or findings from across these methods can address some of the limitations but can also raise additional challenges.

## Online Surveys

Online surveys offer an inexpensive, relatively quick way to invite large numbers of forum users to contribute data on a broad range of questions, which allow statistical analysis of quantifiable outcomes in a way that is convenient for participants and maintains their anonymity. They are less susceptible to social desirability bias and can include data from users who read forums but never post, which is crucial as this subgroup makes up the vast majority of forum users [23]. Surveys can be repeated to investigate change over time. However, surveys need to be designed to accurately assess the concepts being investigated, sensitively worded, and not too arduous to complete. Responders need to be sufficient in number, broadly representative, attentive, anonymous, and consenting. Extensive guidance on survey design already exists (eg, [24,25]), so here we focus only on ethical and practical challenges specific to online surveys evaluating peer online health forums.

### Assessing Important Outcomes

Where possible, health interventions should be evaluated using well-validated health outcome measures. However, such measures are hard to find for the kinds of outcomes that peer support interventions are thought to impact. Rather than reducing physical or mental health symptoms per se, peer support approaches are valued for their positive impacts on acceptance, hope, and normalization [26]. A lack of well-validated measures to use for these concepts, or long burdensome measures, may require evaluators to generate bespoke items for their study, focusing specifically on what they hypothesize will change with forum use.

Where validated measures are used, it is important to screen them for risk-related items and consider how responses on these items will be managed. Online health forums are populated by users who, given their health status, may be at increased risk of harm to self and are also anonymous. If survey respondents complete a survey entirely anonymously, then sharing generic information about how to access support may be sufficient to manage risk. However, for surveys collecting personal details such as email address to send payment or links to follow-up surveys, it may be better to remove specific risk items. Asking users to share limited personal information (an email address) that identifies the individual but does not provide sufficient personal information that would be needed to manage any risk issues (eg, general practitioner details) could be considered unethical.

Surveys are often used longitudinally pre- and postintervention to assess health care impacts. However, forum use often does not have a clear start and end point, as users dip in and out as required. Consequently, analysis of change between “preintervention” and “postintervention” is generally not suitable. If surveys are administered only to new users joining the forum, these will fail to capture the experiences of long-term users, who are often crucial to the functioning of the forum community, unless there is a very long follow-up period. Therefore, surveys often capture a snapshot of activity within the forum. To address this in any analysis, it is important to include items to assess individual forum use patterns, including time since they started to use the forum.

### Facilitating Participation

Surveys in online forums generally rely on convenience sampling. Links to survey items are circulated and promoted among the users, with the aim of recruiting a sufficient and broadly representative sample of users to power statistical analysis to test the study-specific hypotheses. Recruiting and retaining sufficient and representative samples is a big challenge. Studies to date have generally reported low completion rates and lower retention rates for longitudinal surveys, even when financial incentives are used [27]. Methods for promoting the survey can vary between posting adverts in the forum, scrolling advertising banners, and directly emailing or messaging a database of users, all of which will attract the attention of different kinds of users.

Considering careful sampling criteria, providing valued incentives that are not deemed coercive, and standardizing advertising strategies across forums can all help generate adequate samples, the representativeness of which can sometimes be tested, using the demographics collected at forum registration. However, providing incentives can cause problems as they attract the attention of “bad actors” (users who try and take part more than once, people outside of the forum who pretend to be forum users, and bots [autonomous programs on the internet]). More personalized telephone or even face-to-face contact may enhance participation but at the cost of significant resources.

### Validating Participants

Bad actors can be managed by limiting participation to preregistered participants (where these exist), one attempt per email address, and monitoring for suspicious email addresses, such as containing random letters [28] and unlikely patterns of activity (eg, several similar email addresses arriving at the same time [29]). Bots are a particular concern in open forums that can be accessed without first setting up individual accounts. Some survey providers have CAPTCHA (Completely Automated Public Turing test to tell Computers and Humans Apart) built into the software, for example, Qualtrics. However, it should be noted that more recent versions (eg, reCAPTCHA v3) collect personal data without informing the user, which is shared with Google [30], which can be noncompliant with GDPR; and artificial intelligence systems are increasingly able to solve CAPTCHA problems [31].

Even if responders are real people who do meet inclusion criteria, the offer of an incentive may motivate them to take part, but not to attend to the items, resulting in meaningless data. They may also be incentivized to complete the survey multiple times to receive multiple payments. Several strategies exist to screen for this, including looking for unlikely patterns in responding (all items rated at one extreme end), completion times that are impossibly quick, and attention items that are objectively easy and should generate predictable responses if the responder is concentrating. All of these are useful strategies that can be used to “clean” the dataset. However, ethically, incentives still need to be paid to bad actors and suspected bots as it is very difficult to know for sure that this is what they are. Penalizing people who could possibly be genuine responders with attentional problems, which are inherent in many health problems, is unethical. This can result in an expensive dataset and waste of public money.

See iPOF survey details in Textbox 3.

Textbox 3. **iPOF survey.**

**Assessing Important Outcomes**

All forum users aged 16+ were invited to take part in a survey, built in REDCap (Vanderbilt University) [32]. Separate surveys were created, with different links to share in different forums, so it was possible to monitor which forums participants had found the survey from. Survey data were collected between June 2023 and June 2024.

The survey was designed in line with principles proposed by Westthorp et al [33] to directly test our program theories and to sample participants for interview. The survey consisted of 2 well-validated mental health outcomes: Generalized Anxiety Disorder - 7 (GAD-7) [34] and Patient Health Questionnaire - 8 (PHQ-8) [35] plus bespoke items designed to assess specific contextual and mechanistic concepts within the iPOF initial program theories.

The GAD-7 and PHQ-8 assess anxiety and depression and allow comparison with people using the national “NHS Talking Therapies” services program which uses the same measures. The PHQ-8 was used rather than the PHQ-9 because we removed the risk item that assesses suicidal ideation. The survey was used to capture a snapshot of activity and was delivered at 0, 6, and 12 weeks to allow analysis to explore changes over time. The statistical analysis plan was published prior to completing data collection [17].

**Facilitating Participation**

A Participant Information Sheet was designed with extensive host and PPI involvement, to be brief and informative, including signposting participants to additional support resources to manage risk. Inclusion criteria and consent were taken using online check boxes, with the final decision to consent to submit data made at the end, once all items had been completed. An attempt was made to standardize how participants were recruited in each forum, by sharing the same advert wording in each forum. However, some forums also shared the survey by email with registered users to help increase recruitment. Recruitment and retention were incentivized by offering £10 digital shopping vouchers at each time point. Automated emails were used for follow-ups, with up to 2 reminders sent per follow-up.

**Validating Participants**

First, participants gave informed consent to participate in the study. To complete the online consent form, they needed to complete ReCAPTCHA v2 (Google Inc), confirm that they met all inclusion criteria, and enter a valid email address that had not been previously entered. The Participant Information Sheet was available to view here. A total of 2901 consents were received. After consenting, a unique link to the survey was automatically emailed to the participant. A total of 1554 complete surveys were received.

Piloting indicated that attentively completing the survey in less than 5 minutes would be impossible, so all participants who completed the survey in less than 5 minutes were excluded from the data analysis and were not invited to the follow-up surveys. A total of 440 respondents were excluded due to this, leaving a total of 1114.

This group was invited to the follow-up surveys; 576 completed the 6-week follow-up and 532 completed the 12-week follow-up.

After survey data collection was complete, the following variables based on the email addresses of respondents were used to clean the dataset of responses suspected to be from bots or bad actors. Responses were excluded if:

1. the email address domain name was 1 of 8 flagged as being a temporary email address, often used for abuse, by online cybersecurity firms (eg, IPQuality Score [36]) including; Tupanda, Seosnaps, Tospage, Rohoza, Fkcod, Fahih, Laymro, and Oprevolt
2. the email address was very similar (5 or fewer characters difference) to another respondent’s email address submitted to the same forum);
3. the first part of their email address (before the “@”) conformed to a regular pattern of characters that was so common (>250 respondents) we considered it to represent a fraudulent actor making multiple, possibly automated, survey completions. The pattern was: 1 uppercase letter, 3‐12 lowercase letters, 1 uppercase letter, and 3‐12 lowercase letters.

This resulted in a final, cleaned, dataset of 791 participants at baseline, 368 participants at the 6-week follow-up, and 342 at the 12-week follow-up. This compares favorably with previous survey evaluations of online mental health forums that have reported follow-up rates of only 16.6% at 6-week follow-up on similar measures [37].

## Interviews

Interviews offer a way to develop an in-depth understanding of how online forums work through detailed questioning of individuals purposively sampled to address the key questions being asked, and as such are used extensively to evaluate health interventions. As with surveys, people who use forums but never post can also be part of this dataset. However, specific ethical and practical issues arise in interviewing forum users that are concerned with inclusion and anonymity.

### Facilitating Participation

Interviews are generally conducted one-to-one and in-person either face-to-face or via video conferencing. This facilitates rapport and a flexible dynamic approach that can lead to deeper insights into a participant’s experiences and, as such, is very personal. This approach may be particularly unappealing for people who are drawn to forums because of the anonymity and remote asynchronous communication, such as people on the autistic spectrum [38] or who may feel stigmatized by their health condition.

Alternative interviewing styles should be considered that may be more inclusive, including online text-based chat style conversations, which could be done remotely and over longer periods of time. There is some evidence that this can generate high-quality data [39] but can also lead to practical challenges when aiming for a purposive sample if an incentive is offered for taking part. The more remote the interviewee, the harder it is to assess whether they meet the sampling criteria. As surveys, incentives can attract “bad actors” who may pretend to be forum users when they are not.

Language can also be a practical barrier. Even if people are using a forum in which communication is predominantly through one language, this does not mean that they feel confident to take part in an interview in this language. Having the option to involve interpreters for interviews may support people from minority ethnic groups to take part, which is crucial if we are to learn about how forums support health for underserved communities.

### Protecting Anonymity

Care is also needed in sharing and storing data. Interviews are commonly recorded and transcribed, and this should be done using contracted transcribers with relevant confidentiality agreements as part of the research team. Transcripts should be fully deidentified, including removing references to names, places, or the specific health forum, before being stored. In studies in which only one forum is being studied, it can be difficult to hide the identity of the forum, but where multiple forums are being studied, deidentifying each forum, as well as individuals, can greatly reduce the chances of any one individual being recognizable. This should extend to any quotes from participants that are used to illustrate and validate the reporting of analytic findings. Finally, it is not possible to fully anonymize interview data and therefore, the full transcripts should be considered sensitive data and not shared openly.

See iPOF interviews details in Textbox 4.

Textbox 4. **iPOF interviews.**

**Facilitating Participation**

Interviewees were primarily sampled from survey respondents, using a theoretical sampling approach informed by responses on key survey items relevant to the specific program theories being tested. For example, psychological safety is a key feature of forums that determines how users engage with them. We invited users from across the range of responses to a survey item asking how safe they felt on the forum. This allowed us to explore what generated a feeling of safety and what individual and contextual factors were needed to support a sense of safety. Interviews took place between August 2023 and November 2024.

To enhance our inclusive approach, we also purposively sampled people from the forums who had not completed the survey (by advertising directly in the forum space), and people from ethnic minority groups who used forums, or had considered using forums but chosen not to, in order to understand what factors influenced this behavior.

We offered $39 shopping vouchers to all participants, in line with Health Research Authority (HRA) guidance [40]. We were contacted by people we suspected to be bad actors. Some claimed to be from particular demographic groups but this was at odds with their online profiles. Others claimed to have used forums, but then were unable to name any or describe how they worked. We introduced a short screening call using video conferencing in which the interviewer could first gauge demographic characteristics and forum use experience.

Our interview topic guides were informed by realist interviewing methods [41] initially open and inviting participants to share their views about online forums, and gradually funneling to more specific questions to test specific program theories. All topic guides were co-designed with our PPI group, who also offered us the opportunity to practice interviewing and refine the guides. The core elements of the topic guide are available in Multimedia Appendix 1.

**Protecting Anonymity**

Anonymity of participants was ensured through the usual interview procedures of deleting audio data as soon as the file is transcribed and checked; working with a contracted transcriber; and deidentifying transcripts before storage. We took the additional step of deidentifying all forums, by allocating each forum a code name of a UK bird. These codes were used throughout the project to help us keep data linked across different parts of the project, while reducing any risks of identification. The key linking the code to the forum was stored separately from the data. Bird names will be used to refer to forums in all publications and presentations to maintain this secure code. Quotes will be attributed to deidentified individuals within a coded forum.

## Forum Posts

Analyzing forum posts offers a way to see exactly what is happening in forums in real time and without being filtered through the reflections of the participants, or the social desirability and demand characteristics of surveys or interviews. As well as analyzing which topics are being discussed, the metadata attached to forum posts can be used to assess underlying mechanisms of how forums work that members may not be consciously aware of, including the style of language used, speed of responses to posts, etc. Forum post data can also be easy and inexpensive for researchers to collect and free of cost or effort from forum members. However, this method of data collection is ethically and practically the most problematic.

### Gaining Consent

Some forums ask users to consent for their data to be used for research (and often other third-party uses) at registration within the terms and conditions, but use of the forum then depends on them agreeing with these terms. Consequently, this consent is not valid as it is not freely given. Notable exceptions exist in which users are able to opt out of sharing for research and still use the forum, for example, Kooth [42]. This raises different practical (and by implication) ethical concerns about the validity of the data. Posts in forums are generally relational and part of a larger conversation. Analyzing only a subset of these posts from those who consent allows for certain kinds of analyses, for example, topic modeling [43], keyness analysis [44], and sentiment analysis [45] but prevents analysis of the fully contextualized interactions.

Many researchers argue that users in forums in which posts are openly and publicly accessible have no expectation of privacy, and that therefore this can be considered public data not subject to the same GDPR requirements [46]. Thus, much research is done using Reddit (Reddit Inc) and X (formerly known as Twitter; X Holdings Corp). However, this argument has been challenged on the grounds that even if users do not expect privacy of their posts, this does not equate to agreeing to have their data taken out of context and used for other purposes including research [47]. When posts are analyzed by a researcher, they can be interpreted and used to support a viewpoint that the original author may not agree with. Unfortunately, asking for individual consent in open public online health forums is not a solution, as many of the users would never see the request, resulting in the fragmented, decontextualized dataset mentioned above.

A better understanding of public opinion about the use of online health forums for research, and of existing consenting processes in forums, is needed. Some users are likely to want their data to be used for research, or at least some research. A similar public debate about wider sharing of health data without consent suggests that public willingness to share data varies widely depending on the demographics and clinical characteristics of the individual data providers; who the data are being shared with; how well users feel they understand what is being shared and why; the type of data; perceived motivation of the data users; the possible impact of the data user on individual care; how much control individuals feel they have, how much choice, and whether there is a feedback loop so they can see the impacts of data sharing; and financial incentives, especially when data were shared with people making profit from it [48].

Collecting data for research always requires weighing up costs to the individual and society versus the potential benefits to the individual and society. If forums are to play a role in offering support for people with health conditions, then we need to better understand what their impacts are, how they work, and for whom. Forum posts offer a unique way to do this. However, it cannot be at the cost of users feeling unsafe to post in the forums, as psychological safety is fundamental to the continued use and sustainability of forums [15]. This dilemma has led some researchers to focus their analyses on forums that are already closed, for example, Reddit subreddits (eg, [49]), and therefore no risk of disrupting the community. But as Adams et al [47] have argued, inactive forums remove completely the option of users to remove or edit their data. In line with GDPR, we all have the right to be forgotten online [50]. Posts in live forums can generally be edited and deleted by the user, but once a forum is archived or scraped from a website and saved in a database for research, this right is lost. Further, there are no active moderators (forum staff who oversee online conversations) to support collaborative working, and this research could still cause distress to ex-users and impact the use of other online health forums if general trust in forums is eroded.

### Protecting Anonymity

Although often deidentified through use of individually chosen usernames, forum posts are rarely completely anonymous: usernames are often linked to personal details recorded at signup or sometimes reflect the names or identifiable characteristics of an individual; content of posts often refers to people or places that can identify individuals. Users with rare conditions, unusual characteristics, or who hold specific roles, for example, forum moderators, are also at increased risk of being identified. Therefore, forum posts need to be treated as personal sensitive data, with consent needed to share these data for research.

Further important decisions are needed around storage, use, and sharing of forum posts. Storage needs to be done in accordance with the national law, which in the United Kingdom is clearly set out in the GDPR and the Data Protection Act 2018. Forum posts should be deidentified prior to being shared by replacing usernames with a PIN and names of people or places. Deidentified data should be stored in a Secure Research Environment with restricted access and a named researcher who has the responsibility to delete the data after a specified number of years. Forum posts should not be shared as part of the Open Science Framework (OSF [51]). Even when extensive work has gone into deidentifying the dataset, the amount of qualitative data from any one individual increases the risk of users being identified. Sharing the data also removes the right for the person to remove or edit their data (the right to be forgotten), unless the dataset can be accessed and edited by individual users, which would be very difficult but not impossible to implement. Smaller segments of data could be used as quotes in publications to support any forum analysis, but careful paraphrasing needs to be done to ensure these cannot identify individuals by searching for the quote online. For some kinds of qualitative analysis, for example, thematic analysis, this may not be too problematic, but for linguistic analysis, this can alter the characteristics of the data in ways that potentially undermine the analysis itself.

### Collaborative Working

All health research should be a collaborative endeavor with service providers and users. This is particularly crucial for studies using online health forum data where individual consent is not always possible. Decision-making relies on a comprehensive and transparent ethical framework. Close collaboration with forum users, and with hosts and moderators who understand the users of each particular forum is essential to ensure that ethical frameworks are comprehensive and comprehensible [52,53]. Consent given without choice or to very long and incomprehensible terms and conditions documents is not valid. In some instances, this collaborative working may help forums to improve the way in which consent for research is offered.

### Avoiding Function Creep

Analysis of forum data should be limited to the aims of the research for which consent was given by individual users or collaboratively agreed with forum hosts and moderators. PPI at all levels of decision-making, including within the research team and ethics committees, is essential to ensure users are driving what data are used for and to prevent “function creep.” This occurs when researchers use data for purposes beyond the purpose originally approved [54]. Forum posts can create very large datasets, which could be used for a wide range of analyses, and while there are ethical issues in not making the most effective use of data, careful monitoring is needed to ensure researchers are not tempted to use the data for functions beyond their approved use. For example, while consent may have been given for forum posts to be used for some aims, for example, to improve the design of forums for user accessibility, it cannot be assumed that the same consent would have been given for more contentious aims such as to diagnose, predict behaviors, and identify risks, based on algorithms [55].

### Managing Risk

Finally, a challenge in analyzing forum data that is easily overlooked is the risk of exposure to distressing material for the analyst. Some forums contain highly distressing material, some of which, especially in health forums, may have personal relevance for the people analyzing the data. Careful consideration is needed to design an individualized supervision and support plan for everyone in contact with the data. The necessity of this may be obvious to health researchers, especially those with a clinical background, but less familiar to researchers embedded in computational or linguistic disciplines.

See iPOF forum posts details in Textbox 5.

Textbox 5. **iPOF forum posts.**

Forum posts were collected for the period between March 2016 and March 2024.

**Gaining Consent**

During the setup of iPOF, we discussed the study design with the national Confidential Advisory Group (CAG) [56] which provides expert advice on the use of confidential patient information. We were advised that the iPOF study did not require a CAG review as none of the data are considered personally identifiable health data, including forum posts in an NHS-hosted forum in which usernames are linked to patient records. This advice is at odds with the Information Commissioner’s Office which suggests that usernames count as “online identifiers” and should be considered personally identifiable information [57]. This highlights the lack of a clear consensus on what defines identifiable data. We took the more conservative approach and chose to treat all forum posts as potentially identifiable, sensitive data.

For forums in which users can freely give consent at sign-up (ie, they can still use the forum without consenting to this), we used only posts made by consenting users. This resulted in access to 53% of data from one forum. For publicly open forums, we posted about the study onto the forum, with a designated email inviting questions and debate, and we developed in close collaboration with the forum moderators. We gave users the option to email if they wanted their posts removed from the dataset. This led to one user posting that they had checked out our website and found the very detailed outline very reassuring, and there were no further comments or requests for data to be removed. For forums in which consent was not requested at sign-up, or was not freely given, we asked the forum hosts to invite all users to take part by email that included a link to a participant information sheet and an informed consent form. Only data from users who completed this were shared with the research team. In smaller communities, this was easier to do as a greater proportion of the users were still active users and contactable, but this still only led to approximately 4.8% consent. In large forums, this method was not possible due to the higher turnover of users. With one forum, we discussed whether their forum sign-up process could be changed to include consenting for research as an option, rather than a requirement. This did not happen, as the hosts were concerned that this change would reduce the number of people joining the forum, so we could not use any of these forum posts as data. However, the hosts’ decision was at odds with feedback from our PPI group, who felt revising the consent process in this way would enhance trust and increase sign-ups. This is an interesting hypothesis that needs testing. Finally, we considered analyzing posts from forums that have already closed but decided against this as the ethics of this needs further exploration with forum users, which was beyond the scope of iPOF.

**Protecting Anonymity**

All forum datasets were deidentified at the forum and individual levels. Each forum was allocated a UK bird name, and participants were given a PIN to replace their usernames. All names and places were redacted using a combination of manual editing and automatic named entity detection methods. Data were transferred using secure data transfer protocols and stored on secure servers controlled by Lancaster University. The databases will not be openly shared at the end of the study, and all quotes will be paraphrased to reduce the risk of identification of individuals.

**Collaborative Working**

Forum hosts, moderators, and users were embedded throughout the project. Our coapplicant research team included 2 lived experience experts (CL and KM), and a forum moderator (SW). We used a PPI lead (NC) and established a PPI and co-design group. Altogether, 22 PPI participants were recruited, with 13 online forum moderators, 4 forum users, and 5 public advisors. The group met monthly by Zoom for 22 sessions, which were hosted by 2 independent facilitators. Approximately 9 participants and 6 members of the research team attended each session. During the study setup, the group provided feedback on the survey design and interview topic guide, but over time, most sessions were focused on co-designing an animation and a training resource for moderators.

We established a Host group including senior staff involved in forum design and delivery at host organizations. Between 4 and 10 representatives from forum partners attended 5 group meetings, facilitated by PM and FL. Their main role was to advise on the development of forum design guidelines, a key output of the iPOF project. Finally, our Study Steering Group included 2 people with lived experience of using mental health forums, as well as methodologists familiar with the ethical and practical challenges outlined. They met 6-monthly to support the study, including discussion of how to manage the ethical and practical challenges that arose.

**Avoiding Function Creep**

To avoid function creep, the Chief Investigator (CI) was designated as the person responsible for future access to the data, and with responsibility for checking the function of any future use meets current ethical approval, or that further approvals are sought.

**Managing Risk**

Given the potentially distressing and triggering focus of the study, all members of the research team, including those employed for their lived expertise and those employed for their methodological or clinical expertise, were offered a well-being plan. This involved meeting with the PPI lead (CL) to identify possible stressors, likely indicators of stress, and how the individual would like the team to respond to these. Regular supervision with a clinical psychologist (FL) was also offered to staff directly analyzing forum posts and interviewing data.

## Triangulation

All methods outlined above for studying health forums are based on different philosophical assumptions and have inherent strengths and weaknesses that limit the conclusions that can be drawn. Where multiple methods are used, triangulation offers valuable opportunities to integrate the learning from across these methods. Drawing on Denzin [58] and [59], triangulation in the evaluation of health forums can occur at each of 3 levels.

### Triangulating Methods

The first is the methodological level, that is, addressing the same research question by looking at different findings from a range of different methods, for example, Jamison et al [60] analyzed interview transcripts of stroke survivors visiting the general practitioner and forum posts made in an online stroke forum, showing how the 2 data sources complemented the findings from each. Similarly, Thi et al [61] successfully triangulated survey data from pregnant women using a Vietnam-based health service with interview data from other stakeholders in the services to understand mental health help-seeking behaviors. However, triangulating across methods to evaluate online forums can also highlight important philosophical differences. Statisticians may value modeling of survey data collected over time, using standardized questionnaire measures, and assuming that causality can be inferred from examining how changes in one variable precede changes in another; qualitative interviewers may assume that generative causality lies at a deeper level of reality and can only be understood through in-depth interviews to explore how participants react and respond to their experiences within the forum, and corpus linguistics may assume that individuals may not even be aware of how forum use impacts them and that this can best be gleaned from studying subconscious patterns in the language they use. Understanding the value and limitations of each of these approaches is important in realizing the benefits of triangulating them.

### Triangulating Individual Data

The second level of triangulation is the individual participant level, where we can look at data from the same individuals across different methods. This is possible when studying forums, if participants are invited to take part in surveys and interviews through the forum, and the same individuals consent for their forum posts to be analyzed. Additional ethical and practical issues emerge. Additional consent is needed to link these data sources, on top of that given to collect each of the individual sources. The more information that is linked about a person, the greater the chances they become identifiable. In essence, the study moves away from being “issue-centered” toward becoming more “actor-centered” with increased risks for individuals [62]. It is important that participants fully understand the implications of data linkage, and working with a PPI group to find clear ways to communicate this is essential. Triangulating at the individual levels also adds a practical challenge in ensuring that the individual identifiers across the datasets can be matched. For example, in surveys and interviews, participants are often identifiable by name or email address, but in forums, a unique username or PIN is used. Requesting both items of information at the additional consent stage is necessary to ensure the data are linkable.

### Prioritization and Interpretation

Finally, the third level of triangulation is in prioritization and interpretation across different stakeholder groups. Understanding online health forums lends itself very well to interdisciplinary working, stakeholder collaboration, and public and patient involvement, but also highlights significant differences across these groups in what should be prioritized in the findings. This can best be navigated by recognizing these differences at the outset of the study and building in regular opportunities to review the findings and collaboratively agree on what will be taken forward. These can be done online to reduce time, resources, and environmental impact, though some face-to-face collaboration may be needed to build rapport and maintain a collaborative approach across the team. Keeping in mind the aims of the study and the priorities of the funding body can help resolve potential conflict and keep the project on track.

These 3 levels of triangulation are not mutually exclusive, and in the iPOF study, we have attempted to work at all 3, with varying degrees of success.

See iPOF triangulation details in Textbox 6.

Textbox 6. **iPOF triangulation**

**Triangulating Methods**

Realist evaluations encourage the use of any methods that can test the realist theories proposed; however, to date, they have relied heavily on qualitative interview methods [63]. We are still working out how best to triangulate findings from the iPOF survey, interviews, and forum posts. Our experience is that descriptive statistics (context and hypothesized outcomes) from the survey, qualitative analysis of interview data, and linguistic analysis of forum data sit easily within a realist approach, at all stages of theory generation and testing. Hence, we are triangulating these to look at the impacts of forums on mental health outcomes. The practical challenge is publishing them together in an academic journal paper, simply because of the number of words needed to describe all of the methods. The use of statistical models to test realist theories is more challenging. Statistical models often assume linear relationships, are interested in average outcomes, require ever larger numbers to test more complex theories, and aim to show successionist causality. In contrast, our theories are nonlinear, often hypothesize multiple contextual factors triggering multiple mechanisms to generate any one outcome and aim to understand underlying generative causality. We are still working on this challenge.

**Triangulating Individual Data**

Individuals taking part in iPOF consented separately to take part in the survey, interview, or share their forum posts. They could also provide additional consent to have their forum post data linked to their survey data, interview data, or both by providing their username within the forum or email address. This additional consent was explained in a separate participant information sheet, which was developed with our PPI group to ensure it was clear and transparent. Of the 791 valid survey responses, only 105 consented to link these data to forum posts, compared to 478 who consented to be contacted for an interview, and only 18 consented to linking all 3 data sources. This suggests that some triangulation may be more acceptable to participants than others, and that the data are likely to be severely limited.

**Prioritization and Interpretation**

iPOF was funded by the NIHR with a clear focus on the clinical implications of evaluating online mental health forums. This focus was maintained by regular meetings of forum hosts and a PPI and co-design group to codevelop the key outputs including an animation, an e-learning toolkit for moderators, and design guidelines for forums. The theoretical papers outlining the underlying mechanisms by which forums were thought to “work” were developed primarily to inform these clinical outputs, and discipline-specific methodological developments were considered tertiary to this. An independent Study Steering group that met approximately 6-monthly was a helpful guide in steering the project toward the agreed outcomes.

## Discussion

Consistent with our ethos that the perspectives of people with lived experience are essential to any discussion of the ethical and methodological challenges of online research, we first present the following commentary from a forum user and moderator.

### Lived Experience Commentary by Luciana Vega

There are real ethical issues and difficult challenges when considering the vast amount of data in online forums, aiming for meaningful research results. It is reassuring to see here an effort directed at unwrapping different angles and placing a focus on where further consideration might be necessary. It is clear we need to better understand how and why peer support forums work, and an ethical approach is paramount.

I believe the paper explores some challenges well, such as cleaning the dataset from nongenuine users, the difficulties of verifying legitimacy in a largely anonymous, faceless population, and the consent issues of analyzing forum posts. It considers all perspectives on these issues. It would be interesting if the paper had explored the ethical issues when using data from the moderator’s experience too. Also, it only briefly mentioned the idea of a long-term study in a forum specifically designed for data collection, and apart from the funding issue, it would be helpful to have this option further discussed and the ethical issues a “living laboratory” would bring.

I agree with the paper’s suggestion that there is a need to further discuss the ethical costs and benefits of understanding forum designs. Basically, how to balance the ethical priorities versus the need to answer important questions. Any approach that would overcome the main ethical challenges would enable the creation of valuable guidelines for existing platforms. It is accepted that online forums are an easy to access and wide-reaching service that complements other valuable services. However, more research is needed to fully understand what works well and not so well. I believe there is still an urgent need to identify solutions to the main ethical barriers to valid and significant online forum research. This paper suggests some, but more effective solutions are still needed.

### Research Team Commentary

Online forums offer a valuable opportunity for users to share and seek health-related support that is grounded in lived expertise and is easy to access, at a time when health services around the world are struggling to meet demand. Understanding how forums work is important but has inherent ethical and practical challenges. To have ecological validity, research needs to study active forums without disrupting the culture and without relying on highly controlled methods. Impacts for individuals vary widely, are determined by the interactive conversations and therefore are not independent and are not easily measured by standardized measures. There are no clear pre- and postintervention time points, as users dip in and out at will. Evaluation requires triangulating data across a range of qualitative and quantitative methods that are inclusive of users who post in forums as well as those who only read, all of which raise challenges in recruiting and validating participants. Drawing on learning from the iPOF study, we have explored some of the key challenges, including assessing important outcomes, facilitating participation, validating participants, protecting anonymity, gaining consent, managing risk, multistakeholder engagement, and triangulation. iPOF focuses specifically on understanding online forums to support mental health. It may be that forums supporting other health conditions, or other kinds of online spaces, for example, social media platforms, will identify further issues to consider.

Our experiences have highlighted the importance of conducting research in this area, but without risking the trust and psychological safety needed for users to share their personal and sensitive experiences online. The ethical and practical challenges inherent in this are not easily addressed, and the context in which decisions are being made to address these is changing fast. Ethical approval at the outset of a study from a recognized ethics committee is necessary but not sufficient in this context, as many do not have the relevant expertise or connections to scrutinize the changing landscape of societal views. It can be helpful to consider research in this area as an ongoing costs and benefits analysis of the risks of doing the research versus not, but with an awareness that different disciplines and stakeholder groups will have different perspectives that all need to be considered.

We designed iPOF with clear benefits in mind, that is, to develop clinical tools to support hosts and moderators in improving forums for users. We have shared our learning from trying to address the challenges that arose in the hope it will be of value to others designing or reviewing online forum evaluations. We anticipate further risks that we have not yet addressed. Findings may suggest some forum designs are linked to more positive outcomes than others, risking reputational damage for the latter. Concluding that forums can have positive impacts on supporting health could be used to argue for increased funding to support forum delivery, but at the expense of, rather than in addition to, support for other interventions such as talking therapies. Advances in technology and specifically the increased presence of artificial intelligence and bots will fundamentally change the face of online forums, creating new benefits and risks that we cannot yet anticipate.

Having independent steering groups that include a diverse range of stakeholders is essential in all research, but particularly in this area, to ensure that the potential benefits and possible costs for all stakeholders are surfaced and inform decisions being made. Transparency in decision-making throughout the project, including a diverse and fully engaged PPI group, publicly sharing an ethical framework, inviting debate, and learning from previous research, can all help to ensure decisions are accommodating changes in societal context. Structured frameworks such as DECIDE [64] have recently been published to help guide researchers through the key issues to consider in using online data and address many of the issues discussed in this paper. However, further work is needed to maintain safe spaces in which users can openly explore and debate the ethical and practical challenges in evaluating peer online health forums as they evolve technologically and socially to ensure research benefits and avoid harming online health communities that so many users value for the support they offer.

## Supplementary material

10.2196/73427 Multimedia Appendix 1 Interview topic guide.

## Abbreviations

CAG: Confidential Advisory Group
CAPTCHA: Completely Automated Public Turing test to tell Computers and Humans Apart
CCPA: California Consumer Privacy Act
DPIA: Data Protection Impact Assessment
GAD-7: Generalized Anxiety Disorder - 7
GDPR: General Data Protection Regulation
HRA: Health Research Authority
iPOF: Improving Peer Online Forums
NIHR: National Institute for Health and Care Research
OSF: Open Science Framework
PHQ-8: Patient Health Questionnaire - 8
PPI: Patient Public Involvement

## References

[1] Chiauzzi E, Dasmahapatra P, Lobo K, Barratt MJ (2013). Participatory research with an online drug forum: a survey of user characteristics, information sharing, and harm reduction views. Subst Use Misuse.

[2] Bailey E, Robinson J, Alvarez-Jimenez M (2021). Moderated online social therapy for young people with active suicidal ideation: qualitative study. J Med Internet Res.

[3] Smit D, Vrijsen JN, Groeneweg B, Vellinga-Dings A, Peelen J, Spijker J (2021). A newly developed online peer support community for depression (Depression Connect): qualitative study. J Med Internet Res.

[4] (1969). Declaration of Helsinki 1964. WMA.

[5] European Parliament and Council of the European Union (2016). Regulation (EU) 2016/679 of the European Parliament and of the Council. Off J Eur Union.

[6] (2023). Online safety act 2023. UK Government.

[7] Bonta R (2022). California consumer privacy act (CCPA). State of California Department of Justice.

[8] Austin A (2024). Exploring the Archived Web during a Highly Transformative Age.

[9] Lobban F, Coole M, Donaldson E (2023). Improving Peer Online Forums (iPOF): protocol for a realist evaluation of peer online mental health forums to inform practice and policy. BMJ Open.

[10] Gliniecka M (2023). The ethics of publicly available data research: a situated ethics framework for Reddit. Social Media + Society.

[11] Central Digital & Data Office (2020). How to use the data ethics framework. Government Digital Service.

[12] Research Board (2021). Ethics guidelines for internet-mediated research. The British Psychology Society.

[13] (2019). Ethics. AOIR.

[14] Data protection impact assessments. ICO.

[15] Marshall P, Booth M, Coole M (2024). Understanding the impacts of online mental health peer support forums: realist synthesis. JMIR Ment Health.

[16] Lobban F (2023). Ethics framework. Lancaster University.

[17] Shryane N, Glossop Z, Jones S, Lobban F, Marshall P, Meacock R (2024). IPOF survey protocol.

[18] Hariton E, Locascio JJ (2018). Randomised controlled trials – the gold standard for effectiveness research. BJOG.

[19] Lobban F, Akers N, Appelbe D (2020). Clinical effectiveness of a web-based peer-supported self-management intervention for relatives of people with psychosis or bipolar (REACT): online, observer-blind, randomised controlled superiority trial. BMC Psychiatry.

[20] Young C (2013). Community management that works: how to build and sustain a thriving online health community. J Med Internet Res.

[21] Pawson R, Tilley N (2004). Realist Evaluation.

[22] NIHR (2022). Online SoECAT guidance. NIHR.

[23] Wilkerson DA (2016). Lurking behavior in online psychosocial discussion forums: theoretical perspectives and implications for practice. J Technol Hum Serv.

[24] Fielding NG, Lee RM, Blank G (2016). The SAGE Handbook of Online Research Methods.

[25] Ball HL (2019). Conducting online surveys. J Hum Lact.

[26] Cooper RE, Saunders KRK, Greenburgh A (2024). The effectiveness, implementation, and experiences of peer support approaches for mental health: a systematic umbrella review. BMC Med.

[27] Wu MJ, Zhao K, Fils-Aime F (2022). Response rates of online surveys in published research: a meta-analysis. Comput Hum Behav Rep.

[28] Griffin M, Martino RJ, LoSchiavo C (2022). Ensuring survey research data integrity in the era of internet bots. Qual Quant.

[29] Storozuk A, Ashley M, Delage V, Maloney EA (2020). Got bots? Practical recommendations to protect online survey data from bot attacks. Quant Methods Psychol.

[30] (2025). What is reCAPTCHA?. Google.

[31] Plesner A, Vontobel T, Wattenhofer R, Plesner A, Vontobel T, Wattenhofer R Breaking recaptchav2.

[32] Harris PA, Taylor R, Minor BL (2019). The REDCap consortium: building an international community of software platform partners. J Biomed Inform.

[33] Westhorp G, Feeny S (2025). Using surveys in realist evaluation. Evaluation Journal of Australasia.

[34] Spitzer RL, Kroenke K, Williams JBW, Löwe B (2006). A brief measure for assessing generalized anxiety disorder: the GAD-7. Arch Intern Med.

[35] Kroenke K, Strine TW, Spitzer RL, Williams JBW, Berry JT, Mokdad AH (2009). The PHQ-8 as a measure of current depression in the general population. J Affect Disord.

[36] (2025). Detect fraud and cyber threats with unmatched accuracy. IPQS.

[37] Morriss R, Kaylor-Hughes C, Rawsthorne M (2021). A Direct-to-Public Peer Support Program (Big White Wall) versus web-based information to aid the self-management of depression and anxiety: results and challenges of an automated randomized controlled trial. J Med Internet Res.

[38] Yuruki K, Inoue M (2023). Stress and benefits of video calling for people with autism spectrum disorders. PLoS One.

[39] Schiek D, Ullrich CG (2017). Using asynchronous written online communications for qualitative inquiries: a research note. Qual Res.

[40] (2024). Payments and incentives in research.

[41] Manzano A (2016). The craft of interviewing in realist evaluation. Evaluation (Lond).

[42] (2025). Kooth.

[43] Murakami A, Thompson P, Hunston S, Vajn D (2017). ‘What is this corpus about?’: using topic modelling to explore a specialised corpus. Corpora.

[44] Rayson P, Garside R Comparing corpora using frequency profiling.

[45] Wankhade M, Rao ACS, Kulkarni C (2022). A survey on sentiment analysis methods, applications, and challenges. Artif Intell Rev.

[46] Proferes N, Jones N, Gilbert S, Fiesler C, Zimmer M (2021). Studying Reddit: a systematic overview of disciplines, approaches, methods, and ethics. Social Media + Society.

[47] Adams NN (2024). ‘Scraping’ Reddit posts for academic research? Addressing some blurred lines of consent in growing internet-based research trend during the time of COVID-19. Int J Soc Res Methodol.

[48] Baines R, Stevens S, Austin D (2024). Patient and public willingness to share personal health data for third-party or secondary uses: systematic review. J Med Internet Res.

[49] Feldhege J, Moessner M, Bauer S (2021). Detrimental effects of online pro-eating disorder communities on weight loss and desired weight: longitudinal observational study. J Med Internet Res.

[50] (2025). Right to erasure. Information Commissioner’s Office.

[51] (2025). Open Science Framework.

[52] Fiesler C, Beard N, Keegan BC No robots, spiders, or scrapers: legal and ethical regulation of data collection methods in social media terms of service.

[53] Galbraith KL (2017). Terms and conditions may apply (but have little to do with ethics). Am J Bioeth.

[54] Koops BJ (2021). The concept of function creep. Law Innov Technol.

[55] Gooding P, Kariotis T (2021). Ethics and law in research on algorithmic and data-driven technology in mental health care: scoping review. JMIR Ment Health.

[56] (2025). Confidentiality advisory group. NHS Health Research Authority.

[57] What are identifiers and related factors?. Information Comissioner’s Office.

[58] Denzin NK (2017). The Research Act: A Theoretical Introduction to Sociological Methods.

[59] Campbell R, Goodman-Williams R, Feeney H, Fehler-Cabral G (2020). Assessing triangulation across methodologies, methods, and stakeholder groups: the joys, woes, and politics of interpreting convergent and divergent data. Am J Eval.

[60] Jamison J, Sutton S, Mant J, De Simoni A (2018). Online stroke forum as source of data for qualitative research: insights from a comparison with patients’ interviews. BMJ Open.

[61] Thi LM, Manzano A, Ha BTT (2024). Mental health stigma and health-seeking behaviors amongst pregnant women in Vietnam: a mixed-method realist study. Int J Equity Health.

[62] Caliandro A, Gandini A (2016). Qualitative Research in Digital Environments: A Research Toolkit.

[63] Renmans D, Castellano Pleguezuelo V (2023). Methods in realist evaluation: a mapping review. Eval Program Plann.

[64] Shaw H, Brown O, Hinds J, Nightingale S, Towse JN, Ellis D (2023). Describing ethical choices in digital-data explorations (DECIDE)–a toolkit. PsyArXiv.

